# *In vivo* USPIO magnetic resonance imaging shows that minocycline mitigates macrophage recruitment to a peripheral nerve injury

**DOI:** 10.1186/1744-8069-8-49

**Published:** 2012-06-28

**Authors:** Pejman Ghanouni, Deepak Behera, Jin Xie, Xiaoyuan Chen, Michael Moseley, Sandip Biswal

**Affiliations:** 1 Department of Radiology, Molecular Imaging Program at Stanford (MIPS) Stanford, Stanford University School of Medicine, California, USA; 2 Department of Chemistry and Bio-Imaging Research Center, University of Georgia, Athens, GA, USA; 3 Laboratory of Molecular Imaging and Nanomedicine, National Institute of Biomedical Imaging and Bioengineering, Bethesda, MD, USA

**Keywords:** Neuropathic pain, Macrophages, Magnetic resonance imaging, Iron-oxide nanoparticles, Spared-nerve injury model

## Abstract

**Background:**

Minocycline has proven anti-nociceptive effects, but the mechanism by which minocycline delays the development of allodynia and hyperalgesia after peripheral nerve injury remains unclear. Inflammatory cells, in particular macrophages, are critical components of the response to nerve injury. Using ultrasmall superparamagnetic iron oxide-magnetic resonance imaging (USPIO-MRI) to monitor macrophage trafficking, the purpose of this project is to determine whether minocycline modulates macrophage trafficking to the site of nerve injury *in vivo* and, in turn, results in altered pain thresholds.

**Results:**

Animal experiments were approved by Stanford IACUC. A model of neuropathic pain was created using the Spared Nerve Injury (SNI) model that involves ligation of the left sciatic nerve in the left thigh of adult Sprague–Dawley rats. Animals with SNI and uninjured animals were then injected with/without USPIOs (300 μmol/kg IV) and with/without minocycline (50 mg/kg IP). Bilateral sciatic nerves were scanned with a volume coil in a 7 T magnet 7 days after USPIO administration. Fluid-sensitive MR images were obtained, and ROIs were placed on bilateral sciatic nerves to quantify signal intensity. Pain behavior modulation by minocycline was measured using the Von Frey filament test. Sciatic nerves were ultimately harvested at day 7, fixed in 10% buffered formalin and stained for the presence of iron oxide-laden macrophages. Behavioral measurements confirmed the presence of allodynia in the neuropathic pain model while the uninjured and minocycline-treated injured group had significantly higher paw withdrawal thresholds (p < 0.011). Decreased MR signal is observed in the SNI group that received USPIOs (3.3+/−0.5%) compared to the minocycline-treated SNI group that received USPIOs (15.2+/−4.5%) and minocycline-treated group that did not receive USPIOs (41.2+/−2.3%) (p < 0.04). Histology of harvested sciatic nerve specimens confirmed the presence USPIOs at the nerve injury site in the SNI group without minocycline treatment.

**Conclusion:**

Animals with neuropathic pain in the left hindpaw show increased trafficking of USPIO-laden macrophages to the site of sciatic nerve injury. Minocycline to retards the migration of macrophages to the nerve injury site, which may partly explain its anti-nociceptive effects. USPIO-MRI is an effective *in vivo* imaging tool to study the role of macrophages in the development of neuropathic pain.

## Background

Macrophages and other monocyte derivatives, as much as pain-sensing neurons themselves, play a critical role in the generation of chronic pain conditions. Macrophages are derived from the myelo-monocytic stem cells in the bone marrow and play an important role in immunity, inflammation and tissue remodeling. In the setting of nerve injury, macrophages and microglia migrate to various tissues and, as part of the healing response to injury, elaborate a variety of inflammatory factors [1]. In an animal model of peripheral nerve injury, for example, investigators have found that monocytes migrate into the spinal cord, dorsal root ganglia and involved spinal nerve roots, and differentiate into macrophages or microglia [2]. Because of their pro-inflammatory properties, recruitment and activation of monocyte derivatives to the nervous system is thought to promote hypersensitivity and chronic pain states.

An *in vivo* magnetic resonance imaging (MRI)-based method using magnetic nanoparticles, such as superparamagnetic iron-oxide particles (SPIOs), ultrasmall SPIOs (USPIOs), monocrystalline iron-oxide particles (MIONs) and cross-linked iron oxide (CLIO) has been developed to track macrophage and T-cell migration and localization [3]. Ultrasmall superparamagnetic iron-oxide magnetic resonance imaging (USPIO-MRI) allows monitoring of trafficking of macrophages into the central nervous system in a variety of degenerative neurological conditions [4]. SPIOs have also been used to monitor monocytic/macrophage migration patterns in the setting of rheumatoid arthritis. After intravenous injection of SPIO particles, cells that reside in the reticuloendothelial system (RES), including macrophages, engulf the agent. Because macrophages are recruited to inflamed joints, monitoring their distribution by SPIO-based techniques can be helpful, especially during early phases of the disease. MRI can be used to study the migration of these cells from the RES to inflamed joints. Investigators have successfully documented the migration of SPIO-labeled macrophages to the synovium of a rat model of RA [5].

Another method to monitor T-cell traffic has been developed for MRI. T-cells isolated from a subject can be loaded with dextran-coated SPIO or similar dextran-coated CLIO [6,7]. When exposed to SPIO, T-cells will engulf the 30 nm particles by endocytosis. The T-cells are eventually re-introduced into the subject, and the subject is scanned. On gradient-echo sequences, cells carrying this contrast agent appear low in signal intensity owing to the large susceptibility effect generated by the sequestered SPIO particles. In rat models of cardiac, renal and lung allograft rejection, migration of SPIO-labeled T-cells to the allograft has been found during rejection [8-10].

Using USPIO-MRI as a surrogate marker for macrophage recruitment, we sought 1) to detect nociception-related spatiotemporal USPIO-MRI signal changes in a peripheral nerve after injury *in vivo-* in a longitudinal animal model of pain, 2) to determine whether chronic pain states correlate with macrophage recruitment, and 3) to determine whether USPIO-MR can be used to monitor the known effect of the antibiotic minocycline on macrophage trafficking to the site of nerve injury and whether this in turn results in altered pain thresholds.

## Results

### Minocycline affects pain behaviors

**Figure 1:**
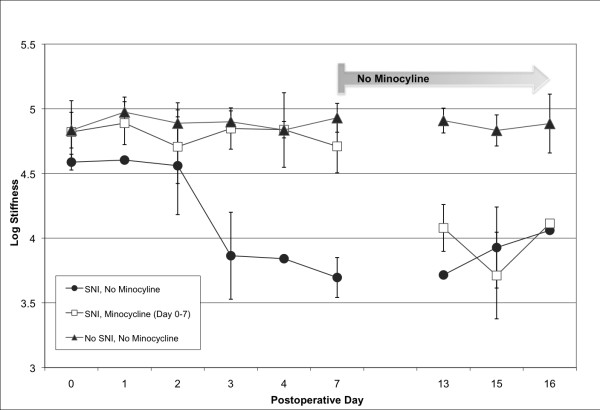
**Minocycline administration prevents development of mechanical allodynia after peripheral nerve injury.** Mechanical sensitivity was assessed by the von Frey test before, and 1, 3, 7, 13, 15, and 16 days after nerve injury. The 50% response threshold of the injured left hindpaw decreased to 3.86 ± 0.34 by 72 hours (squares). The response threshold of rats receiving daily intraperitoneal injections of minocycline did not change (4.90 ± 0.08, triangles) and was similar to the threshold of the uninjured rats (4.85 ± 0.16, triangles, red line). Minocycline administration was stopped after seven days; one week later, all injured rats demonstrated the same threshold. Averages and standard deviations of data collected from 3 – 6 animals at each time point are presented.

Minocycline is known to prevent allodynia in both inflammatory and mechanical nerve injury models, and has been shown to decrease macrophage recruitment after nerve injury [1]. Before testing the impact of this drug on macrophage trafficking by MR, we first confirmed minocycline’s ability to prevent allodynia after sciatic nerve injury in our model of neuropathic pain. Behavioral measurements confirmed the presence of allodynia in the neuropathic pain model (50% paw withdrawal threshold of 3.86 ± 0.34) while the paw withdrawal threshold of the minocycline-treated injured group was significantly higher (4.90 ± 0.08, p < 0.011), and was similar to the threshold of the uninjured paw (4.85 ± 0.16). Without minocycline, the injured rat demonstrated increased sensitivity to previously innocuous stimuli within 72 hours after injury, reaching a plateau within one week. In order to determine the persistence of the effect of the drug, minocycline administration was then stopped at this point, one week after injury. One week after termination of minocycline treatment, all injured rats demonstrated the same level of allodynia (3.90 ± 0.17 for previously minocycline treated rats compared to 3.97 ± 0.22 for the untreated rats, Figure 1).

### Minocycline alters trafficking of USPIO-laden macrophages

**Figure 2:**
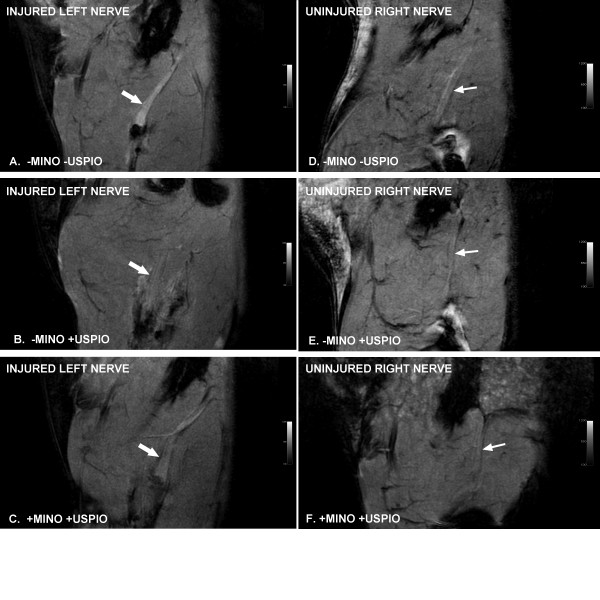
**Representative sagittal FIESTA images of the left and right thighs and sciatic nerve of rats with neuropathic pain obtained 7 days following spared nerve injury (SNI).** White arrows indicate location of sciatic nerve proximal to the nerve injury. +MINO indicates the animal received daily intraperitoneal minocycline injections. +USPIO indicates the animal received an intravenous dose of USPIOs immediately after peripheral nerve injury and on the day prior to MRI. This dose and timing of administration has been shown to label macrophages. The low signal at the distal truncated end of the nerve corresponds to the site of ligature. A) SNI rat that received neither minocycline nor USPIOs. The sciatic nerve demonstrates increased signal intensity relative to the surrounding muscle. The injured nerve was enlarged compared to the uninjured side (data not shown). B) SNI rat that received USPIOs but not minocycline. There is iron-induced signal loss, as the sciatic nerve is nearly isointense to the surrounding muscle. C) SNI rat that received both minocycline and USPIOs. The signal intensity in the sciatic nerve is higher than with USPIOs alone, suggesting that minocycline retards the migration of USPIO-laden macrophages to the site of injury.

**Figure 3:**
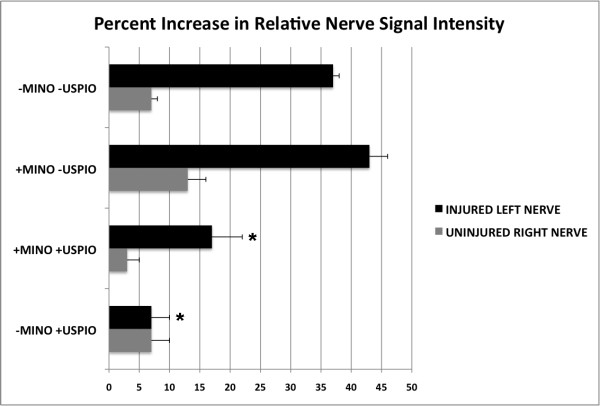
**Percent increase in relative nerve signal intensity in the setting of minocycline and USPIO treatment.** The signal intensity of the injured left and uninjured right sciatic nerve proximal to the site of injury was normalized to that of the adjacent muscle. Asterisks denote injured *left* nerve treatment groups that were significantly different from the untreated control animals (Bonferroni’s post hoc multiple comparison test, p < 0.05 for +USPIO versus +MINO +USPIO; p < 0.001 for +MINO +USPIO and for +USPIO versus -MINO -USPIO; no difference between +MINO and –MINO -USPIO). The signal intensity of uninjured right nerve was only mildly higher than that of surrounding muscle (3 to 13%), which was independent of minocycline and/or USPIO administration. Data represent the averages and standard deviations of three animals in each group.

We then sought to monitor minocycline’s effect on macrophages by labeling these cells *in vivo* using ultrasmall superparamagnetic iron oxide particles. After intravenous injection, these particles are phagocytosed by reticuloendothelial cells, including macrophages [2]. Delivery of the particles was confirmed by abdominal MR imaging that demonstrated a dramatic decline in the T2 weighted signal intensity of the liver and spleen in animals injected with USPIOs. On FIESTA MR, the injured sciatic nerve was enlarged compared to the uninjured nerve, and demonstrated increased signal intensity relative to muscle (relative MR signal intensity: 37 ± 1%, Figure 2A). Minocycline treatment alone did not alter the increased signal intensity of the injured nerve (43 ± 3%). However, with USPIO administration, the injured nerves became nearly isointense to the surrounding muscle (7 ± 3%, Figure 2B), suggesting that USPIO-laden cells had migrated to the site of injury. While this decline in signal was specific to animals that received USPIO injections, we further assessed for post-operative hematomas as a possible confounding source of iron-laden cells. No hematoma was evident at the site of injury during the course of imaging. In addition, copious saline washes of the surgical site did not alter the MR signal properties of the injured nerve (data not shown). Administration of minocycline significantly reduced the USPIO effect (nerve signal relative to muscle: 17 ± 5%, Figure 2C, Figure 3, p < 0.05). In order to determine the etiology of the decreased MR signal of the injured nerve in rats treated with USPIOs, rat sciatic nerves were harvested and examined. Of note, the signal intensity of uninjured right nerve was only mildly higher than that of surrounding muscle (3 to 7%), which was independent of minocycline and/or USPIO administration (Figures 2F, Figure 3).

### Histologic presence of iron-laden macrophages at the site of nerve injury is dependent upon minocycline treatment

**Figure 4:**
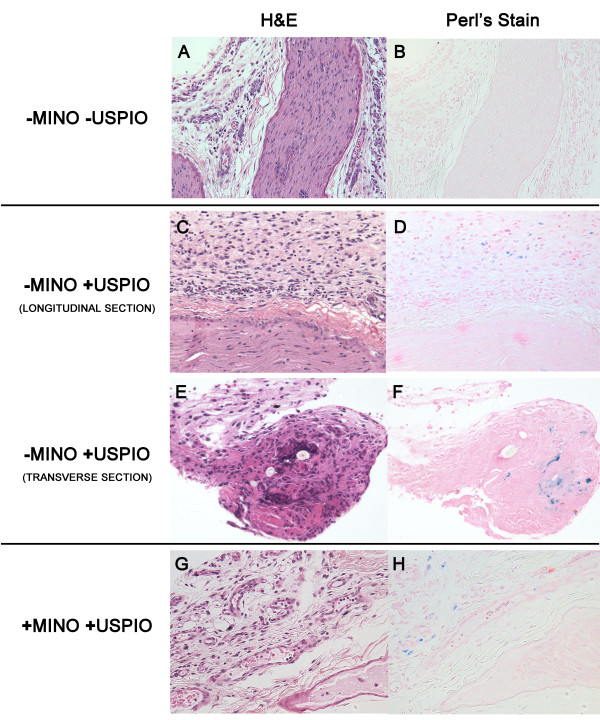
**Histology verifies MR imaging findings with perineural iron-laden macrophages demonstrated only in animals injected with USPIOs without minocycline.** Paraffin sections were prepared from the distal segment of the nerve, just proximal to the ligature, and stained with (A, C, E, G) hematoxylin and eosin (H&E) to provide morphological detail and with (B, D, F, H) Perl’s stain, which stains iron blue. (**A**, **B**) Control (−USPIO) longitudinal sections through the neuroma and surrounding perineural tissue harvested from the site of the neuroma show no iron staining. (**C**-**F**) Tissues harvested from SNI animals injected with USPIOs. At 200X magnification, the axial section and the longitudinal section (**C**, **D**) and an axial section (**E**, **F**) from a different animal show areas of increased iron staining in the perineural tissues. These iron containing cells demonstrate histologic characteristics of macrophages. At 200x magnification, iron and H&E stained axial sections from animals treated with minocycline and USPIOs (+MINO-USPIO) show localization of iron containing cells to the perineural tissue, albeit to a qualitatively lesser extent than the –MINO+USPIO treated sections.

Histology of these harvested sciatic nerve specimens confirmed the presence of iron-laden macrophages at the nerve injury site only in the group of injured rats that received USPIOs without minocycline treatment (Figure 4C-F). By comparison, rats that received minocycline had qualitatively fewer iron-laden macrophages at the site of nerve injury as determined by Perl’s staining (Figure 4G, H).

## Discussion

After nerve injury, Schwann cells generate inflammatory chemoattractants that recruit macrophages to the site of nerve injury, where these cells initiate an inflammatory cascade involving release of cytokines such as monocyte chemoattractant protein-1 (MCP-1), interleukins, and nerve growth factor as part of the process of Wallerian degeneration [11]. However, both centrally and peripherally, this inflammatory process can result in the development of neuropathic pain [2].

For example, mutations in mice that result in delayed or absent macrophage activation, such as knockouts of the chemokine receptor chemotactic cytokine receptor 2 (CCR2), have impaired neuropathic pain responses [12]. Alternatively, MCP-1 upregulation in the spinal cord is associated with increased pain hypersensitivity [13]. B7-H1 (−/−) mice, which possess enhanced macrocytic and T cell responses to injured peripheral nerves, exhibit more pronounced mechanical hyperalgesia [14]. Minocycline prevents the development of allodynia, and retards the migration of macrophages to the nerve injury site. The mechanism of minocycline action may be due to inhibition of matrix metalloproteases and inhibition of angiogenic factors, thereby preventing breakdown of the blood-nervous system barrier and revascularization [15].

In our study, animals with neuropathic pain in the left hindpaw show increased migration of USPIO-laden macrophages to the site of sciatic nerve injury, suggesting that USPIO-MRI is an effective tool to study the role of macrophages in the development of neuropathic pain. If we could take a picture of an individual’s pain-sensing circuits and highlight which foci are associated with abnormal macrophage infiltration/microglia activation, this would be invaluable in helping to objectively diagnose an individual’s cause of chronic pain. Our ability to correlate minocycline’s effect on the development of neuropathic pain with the effect of minocycline *in vivo* on macrophage recruitment suggests a role for this imaging tool in pre-clinical development of new drugs for pain control or in facilitating clinical image-guided therapies. Although currently not commercially available, iron oxide-based MRI has already been developed and approved for human use; Feridex^©^and Combidex^©^ (feroxumide injectable solutions, AMAG Pharmaceuticals, Inc., Cambridge, MA) have been available for clinical use, for example. Identifying patients with macrophage infiltration or microglial activation along nociceptive pathways in the nervous system will help localize and characterize chronic pain pathways and could promote the use of analgesic regimens that include macrophage inhibitors such as minocycline.

## Conclusions

Animals with neuropathic pain in the left hindpaw show increased trafficking of USPIO-laden macrophages to the site of sciatic nerve injury. Minocycline appears to retard the migration of macrophages to the nerve injury site, which may partly explain its anti-nociceptive effects. USPIO-MRI is an effective tool to study the role of macrophages *in vivo* in the development of neuropathic pain and, thus, may aid in the development of novel analgesics and has the potential to be used to study macrophage behavior and localization in living human subjects who suffer from chronic pain.

## Methods

### Animal models

Experiments were carried out using adult Sprague–Dawley rats weighing 200-250 g. The Peripheral Nerve Injury Model (Neuropathic Model), otherwise known as the ‘spared nerve injury’ (SNI) model, served as a model of persistent peripheral neuropathic pain [16]. Briefly, under isoflurane anesthesia, the rat left sciatic nerve was exposed above the trifurcation, and then the tibial and common peroneal nerves were isolated, ligated, and cut, leaving the sural nerve intact. These animals develop hyperalgesia and allodynia within ~3 days after injury.

### Ultrasmall paramagnetic iron oxide (USPIO) particle preparation

Oleate coated iron oxide nanoparticles (15 nm) were obtained from Ocean NanoTech (Springdale, AR). For surface modification, about 50 mg of oleate coated nanoparticles were dispersed in 10 ml of chloroform. Forty milligrams of dopamine in 10 ml DMSO were added to the solution. The mixture was heated at 70 °C for 1 hour, and then cooled down to room temperature. Hexane was used to precipitate the nanoparticles, which were then centrifuged at 15,000xg for 15 minutes, dried with nitrogen, and redispersed in DMSO via sonication. The nanoparticles were added to a solution of 50 mg HSA in borate buffer (50 nM, pH 8.5), and dialyzed against PBS buffer (pH 7.4) for 24 h. The nanoparticles were centrifuged at 30,000 g for 20 minutes to remove the free HSA, and were redispersed in PBS buffer. Small aggregates were removed by passing the particles through a syringe filter (0.22 μm).

### USPIO-magnetic resonance imaging (MRI) and minocycline treatment

All MRI experiments were performed on a small animal imaging unit (7.0 T Magnex; GE Medical Systems) located in the Small Animal Center for *In-Vivo* Imaging in the Clark Center at Stanford University School of Medicine. Animals were anesthetized with humidified, oxygen-enriched 2-4% isoflurane administered via nose cone. Animals were placed in a rodent holder and then into a rat quadrature body coil. Temperature and respiration were monitored during the imaging procedure. While under anesthesia and immediately after nerve injury, animals received an intravenous USPIO injection (300 μmol/kg IV; mean particle size of 20 nm) with or without minocycline (50 mg/kg IP QD, Sigma-Aldrich, St. Louis, MO). Additional doses of USPIO were administered 24 hours prior to MR imaging. Animals were imaged at post-operative day 7. A FIESTA (Fast Imaging Employing Steady State Acquisition) sequence, with TE/TR/FA of 11/22/20, slice thickness of 1 mm and in plane resolution of 0.11 x 0.11 mm was used. Using Osirix image analysis software, regions of interest were circled on bilateral sciatic nerves to quantify signal intensity, which was then normalized to background signal in the muscle.

### Pain behavior measurements

A logarithmic series of calibrated monofilaments (Von Frey hairs; Stoelting, Wood Dale, IL) were applied to the left and right hindpaws to determine the threshold stiffness required for 50% paw withdrawal. Log stiffness of the hairs is defined as log_10_(milligrams x 10) and ranged from 3.61 (407 mg) to 5.18 (15,136 mg). The log stiffness that would have resulted in a 50% paw withdrawal rate was computed by fitting a Gaussian integral psychometric function to the observed withdrawal rates for each of the tested von Frey hairs using a maximum likelihood fitting method that allows parametric statistical analysis.

### Histology

Extracted intact sciatic nerves proximal to the site of ligature were harvested after the animal was euthanized on day 7 after imaging. Samples were fixed and stained either with hematoxylin and eosin, or with Perl’s stain for iron per standard protocols.

### Statistical analysis

Data were analyzed by one-way ANOVA performed on GraphPad Prism (San Diego, CA), with p value <0.05 accepted as significant.

## Abbreviations

MRI: Magnetic resonance imaging; USPIO: Ultrasmall paramagnetic iron-oxide; SPIO: Superparamagnetic iron-oxide; MINO: Minocycline; FIESTA: Fast imaging employing steady state acquisition.

## Competing interests

The authors declare that have no competing interests.

## Authors' contributions

PG and DB carried out all of the animal studies, including surgery, pain behavior assessments and imaging. They also performed the statistical analysis. JX and XC prepared the USPIOs. SB conceived of the study, participated in its design and coordination. PG, DB, MM and SB helped draft the manuscript. All authors read and approved the final manuscript.

## References

[B1] Scholz J Woolf CJ The neuropathic pain triad: neurons, immune cells and glia Nat Neurosci 2007 10 1361 1368 10.1038/nn1992 17965656

[B2] Watkins LR Maier SF Beyond neurons: evidence that immune and glial cells contribute to pathological pain states Physiol Rev 2002 82 981 1011 12270950 10.1152/physrev.00011.2002

[B3] Thorek DLJ Czupryna J Chen AK Tsourkas A Hayat MA Molecular Imaging of Cancer with Superparamagnetic Iron Oxide Particles Cancer Imaging: Instrumentation and Applications 2008 Elsevier Acad Press, San Diego 85 95

[B4] Stoll G Bendszus M Imaging of inflammation in the peripheral and central nervous system by magnetic resonance imaging Neuroscience 2009 158 1151 1160 10.1016/j.neuroscience.2008.06.045 18651996

[B5] Beckmann N Falk R Zurbrugg S Dawson J Engelhardt P Macrophage infiltration into the rat knee detected by MRI in a model of antigen-induced arthritis Magn Reson Med 2003 49 1047 1055 10.1002/mrm.10480 12768583

[B6] Dodd SJ Williams M Suhan JP Williams DS Koretsky AP Ho C Detection of single mammalian cells by high-resolution magnetic resonance imaging Biophys J 1999 76 103 109 10.1016/S0006-3495(99)77182-1 9876127 PMC1302504

[B7] Josephson L Kircher MF Mahmood U Tang Y Weissleder R Near-infrared fluorescent nanoparticles as combined MR/optical imaging probes Bioconjug Chem 2002 13 554 560 10.1021/bc015555d 12009946

[B8] Kanno S Lee PC Dodd SJ Williams M Griffith BP Ho C A novel approach with magnetic resonance imaging used for the detection of lung allograft rejection J Thorac Cardiovasc Surg 2000 120 923 934 10.1067/mtc.2000.110184 11044319

[B9] Kanno S Wu YJ Lee PC Dodd SJ Williams M Griffith BP Macrophage accumulation associated with rat cardiac allograft rejection detected by magnetic resonance imaging with ultrasmall superparamagnetic iron oxide particles Circulation 2001 104 934 938 10.1161/hc3401.093148 11514382

[B10] Zhang Y Dodd SJ Hendrich KS Williams M Ho C Magnetic resonance imaging detection of rat renal transplant rejection by monitoring macrophage infiltration Kidney Int 2000 58 1300 1310 10.1046/j.1523-1755.2000.00286.x 10972694

[B11] Martini R Fischer S Lopez-Vales R David S Interactions between Schwann cells and macrophages in injury and inherited demyelinating disease Glia 2008 56 1566 1577 10.1002/glia.20766 18803324

[B12] Abbadie C Lindia JA Cumiskey AM Peterson LB Mudgett JS Bayne EK Impaired neuropathic pain responses in mice lacking the chemokine receptor CCR2 Proc Natl Acad Sci USA 2003 100 7947 7952 10.1073/pnas.1331358100 12808141 PMC164693

[B13] Gao YJ Zhang L Samad OA Suter MR Yasuhiko K Xu ZZ JNK-induced MCP-1 production in spinal cord astrocytes contributes to central sensitization and neuropathic pain J Neurosci 2009 29 4096 4108 10.1523/JNEUROSCI.3623-08.2009 19339605 PMC2682921

[B14] Uceyler N Gobel K Meuth SG Ortler S Stoll G Sommer C Deficiency of the negative immune regulator B7-H1 enhances inflammation and neuropathic pain after chronic constriction injury of mouse sciatic nerve Exp Neurol 2010 222 153 160 10.1016/j.expneurol.2009.12.026 20051242

[B15] Ledeboer A Sloane EM Milligan ED Frank MG Mahony JH Maier SF Minocycline attenuates mechanical allodynia and proinflammatory cytokine expression in rat models of pain facilitation Pain 2005 115 71 83 10.1016/j.pain.2005.02.009 15836971

[B16] Decosterd I Woolf CJ Spared nerve injury: an animal model of persistent peripheral neuropathic pain Pain 2000 87 149 158 10.1016/S0304-3959(00)00276-1 10924808

